# Nurses’ Experience of Nursing Workload-Related Issues during Caring Patients with Dementia: A Qualitative Meta-Synthesis

**DOI:** 10.3390/ijerph181910448

**Published:** 2021-10-04

**Authors:** Younhee Kang, Yujin Hur

**Affiliations:** 1 Division of Nursing, College of Nursing, Ewha Womans University, Seoul 03760, Korea; yxk12@ewha.ac.kr; 2 Graduate Program in System Health and Engineering, Ewha Womans University, Seoul 03760, Korea

**Keywords:** dementia, Alzheimer’s disease, nurses, workloads, work-related stress, qualitative research

## Abstract

The behavioral and psychological symptoms of dementia (BPSD), which appear in all dementia patients, demand sizable commitments of time and effort from nurses. This study aims to identify issues related to the workloads of nurses who provide care for dementia patients via qualitative meta-synthesis. Eleven articles were selected using a systematic review flowchart, which were then evaluated for their quality using the Critical Appraisal Skills Program checklist. Collected data were analyzed using a line-of-argument method. Theme clusters were “increased workload due to characteristics of dementia”, “increased mental stress”, “difficulty associated with playing a mediator role in addition to nursing duties”, and “lacking systematic support for dementia patient care”. To reduce the workload and mental stress of nurses in dementia care, supportive measures appropriate for their occupational characteristics should be developed, based on workload estimates that account for the attributes of dementia patients.

## 1. Introduction

Currently, the large growth in the number of the elderly, correlated with improvements in life expectancy, have led to increased prevalence rates of chronic diseases such as dementia. In 2018, the number of dementia patients worldwide was approximately 50 million, 1.06 times greater than three years ago; in 2015, the number was 46.8 million [1]. At this rate, the number of dementia patients will reach approximately 131.5 million by 2050 [1]. Along with the rise in the number of patients, the medical expenditures for elderly dementia patients is also increasing annually, growing from 277 billion USD in 2018 to 290 billion USD in 2019 [1,2]. In addition, the total maintenance cost of dementia—which includes caregivers’ lost working hours and health maintenance fees, along with the direct expenses of dementia treatment—was estimated to be around 818 billion USD in 2015 [3]. Thus, dementia results in socioeconomic losses for families and local communities. In addition, its impact is manifested in various forms, based on the socioeconomic status of the affected individuals, reflecting the socioeconomic inequality of health. Prevalence rates of dementia differ by ethnicity, education level, economic standard, and residential district [4]. In the United States, Hispanic and Black populations have indicated higher dementia prevalence rates compared with White populations; dementia prevalence rates are reported to be higher for lower educational levels and socioeconomic status as well [4,5,6].

The behavioral and psychological symptoms of dementia (BPSD), which include neuropsychiatric symptoms of delusion, hallucination, aggression, depression, impassivity, and abnormal behavior, appear in all dementia patients; for almost half of all patients, BPSD appear at critical levels [7,8]. Such neuropsychiatric symptoms increase patients’ fall risk and render it necessary for nurses to constantly monitor them, as they can be potentially hazardous for other patients [9]. The BPSD of dementia patients can even cause negative emotions for nurses, such as frustration, anxiety, fear, and sadness [10]. In addition to the complex demands of dementia patients, nurses must ascertain the needs of the patients’ guardians, and strike a balance between the two [11]. Thus, there are numerous demands required of nurses who care for dementia patients, thereby leading to increased workloads [9].

The hospitalization rate of dementia patients is 1.49 times that of other patients [12], and the 30-day readmission rate is reported to be 7–35% [13]. In addition, as 23% of dementia patients experience long-term hospitalization of 180 days or more [14], nurses in dementia care are tasked with significant responsibilities. Dementia patients demand more time and commitment from nurses due to their neuropsychiatric symptoms [10,15]. Particularly, types of nursing care for patients with dementia vary from the basic bedside nursing care such as personal hygiene, emotional supports (being a companion to talk with), drug therapy, and safety care, to specific dementia care including cognitive therapy, rehabilitation, and other diverse therapies [16,17].

As nurses maintain a close relationship with the patient and provide holistic care, they can recognize the cognitive change process of the patient and corporate care with other chronic diseases [16]. The care delivered by nurses has been reported to affect the overall quality of treatment [17]. However, some factors hinder nursing care for patients with dementia, such as lack of knowledge and experience [18] or helplessness [19]. Therefore, to increase the quality of care provided to dementia patients, it is necessary to identify issues related to the nursing for dementia patients, by exploring the experiences of nurses in dementia care.

The purpose of this study is to comprehensively explore nurses’ experiences in providing care for dementia patients via a meta-synthesis of qualitative research related to occupational issues experienced by nurses in dementia care. Ultimately, the goal of this study is the comprehensive identification and understanding of nursing workload-related issues for dementia patients. This paper reports on the issues of the general type of nurses, not those referred to as NPs or dementia specialist nurses, to identify barriers and inhibitors of general care.

## 2. Methods

This study is a qualitative meta-synthesis that analyzes the results of qualitative research articles that explore nurses’ experiences of providing care for dementia patients.

### 2.1. Data Collection Methods

The study has selected articles based on the systematic review flowchart of the Preferred Reporting Items for Systematic Reviews and Meta-Analyses (PRISMA) [20].

#### 2.1.1. Literature Search and Article Selection

The following databases were used to find qualitative research articles on nurses’ experiences of providing care for dementia patients: Pubmed, CINAHL, PsycINFO, and Research Information Sharing Service (RISS). Keywords were set to “dementia (MeSH)” or “Alzheimer disease (MeSH)” or “dementia” or “Alzheimer” AND “nurses (MeSH)”or “nurse” or “nursing” or “nurs*” with the time period set from 2011 to 2021, in order to search for articles published in the past decade. Specifically, the search of studies in Pubmed and CINAHL databases were performed, including MeSH terms, and the RISS database was searched in Korean. Eligibility criteria were (a) qualitative studies, (b) dealing with general registered nurses’ experiences of caring patients with dementia, and (c) published to peer-reviewed journal for the validity of our study. Exclusion criteria were (a) articles that also analyze experiences of occupations other than nurses, to focus on nurses’ experiences, (b) articles that analyze the experiences of specific nurses (e.g., nurse practitioner, admiral nurse, or nurse manager), to focus on general nurses’ experiences, (c) articles in languages other than Korean and English, and (d) articles with an exclusive focus on specific dimensions of dementia care (e.g., end-of-life care, spiritual care) as it just gives us specific information. To select articles, each researcher individually evaluated the article and related content and proceeded to check for consensus. When there were disagreements or uncertainties, the researchers thoroughly discussed and consulted with each other before making the final decision on selecting an article.

**Figure 1:**
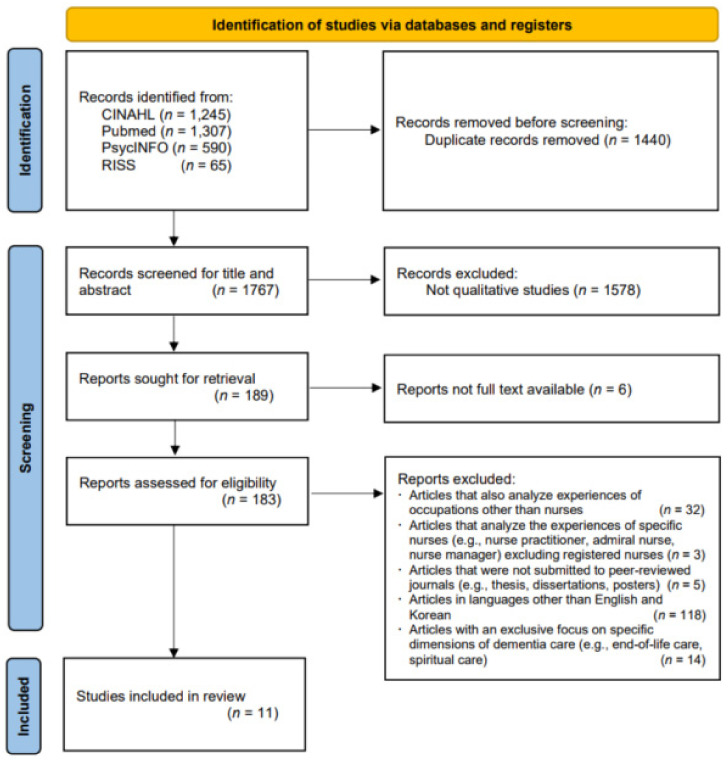
Flowchart of the Preferred Reporting Items for Systematic Reviews and Meta-Analyses.

In total, 3207 articles were extracted from the initial search; 1767 article titles and abstracts were reviewed, with the exclusion of 1440 overlapping articles. After applying the selection and exclusion criteria, a total of 15 articles were reviewed for their full text, and 1747 articles were excluded. In the full text screening, four articles that included personnel other than registered nurses (e.g., social workers or certified nursing assistants) were also excluded, leaving 11 research articles as the focus of the final analysis (see Figure 1).

#### 2.1.2. Quality Appraisal

The quality of the selected articles was evaluated using the Critical Appraisal Skills Program (CASP) checklist for qualitative research [21]. The resulting quality appraisal scores ranged from a minimum of 5 to a maximum of 9. As the quality appraisal results are more appropriate as a supplement for understanding articles than as a selection criterion for a qualitative research meta-synthesis approach [22], the study did not exclude articles based on the quality appraisal results. There were no articles that met all items of the CASP checklist; for instance, some articles did not address potential researcher bias and participant recruitment strategies. However, articles that remained in the final selection were all peer-reviewed, published in reputable academic journals, and included quality information on nurse experiences in dementia care [23,24,25,26,27,28,29,30,31,32,33]; therefore, they were deemed to have ample value as data for the current study.

### 2.2. Data Analysis

The line-of-argument synthesis method from Noblit and Hare’s meta-ethnography was used for data analysis [34]. This method aims to synthesize the core components of qualitative studies by exploring the similarities and differences of selected articles. It aspires toward a deeper understanding of research phenomena, finding new viewpoints and identifying the core component of the phenomenon of interest by comparing, contrasting, merging, and synthesizing different studies.

First, themes were identified and organized from the results sections of each study, with detailed comments on the meaning of each theme. Using the CASP checklist scores and publication years, the themes and subthemes of each article were ordered. The article with the highest CASP checklist score, Fatania et al. [23], was analyzed thoroughly. Then, another article [24] was analyzed thoroughly by comparing the meaning of topics and contexts of the first analysis article. By repeating this process multiple times, broad and overarching concepts were enriched with more concrete details, becoming more specific concepts. To re-evaluate the results of this process, themes and meanings were cross-evaluated with the themes and meanings of the original article. The re-evaluated results were then synthesized under a shared concept, which were named accordingly to represent new concepts. Finally, the newly named concepts were combined under superordinate concepts that incorporate them, which were also named accordingly.

### 2.3. Rigor

To ensure the rigor of the data analysis, the standards suggested by Lincoln and Guba were used [35]. Meta-analysis research rigor comprises four constituents: truth value, applicability, consistency, and neutrality [35]. To ensure truth value, data from the selected articles were presented as reported. Researchers cross-checked the original text of the selected articles with the resulting synthesis of the results multiple times, ensuring that the articles were reflected accurately while also checking for citation errors. Applicability is the consistency between the results and discussion of the meta-analysis research and the real-life applications of such findings. As the current study analyzes the occupational experiences of nurses in dementia care, it can affect the nursing duties of nurses undertaking such care. Thus, the researchers checked whether nurses’ clinical phenomenology was reflected in selected articles, with clinical experience and continued research experiences in related topics. The constituent of consistency evaluates whether the results have been derived logically through the research and analysis process. The researchers re-examined whether the research design and analysis interpretations have been clearly outlined and decided upon. To ensure neutrality, the researchers made conscious efforts to exclude individual prejudices and experiences and prevent their intervention in the analysis process, analyzing the data from the most objective perspective possible.

## 3. Results

The selected articles are shown in Table 1, which outlines the purpose of the study, participants, method of analysis, results, and results of the quality appraisal. A total of 181 participants were included in the review, and most of the participants worked in long-term care facilities. The included articles were from five countries.

A total of four themes were synthesized from the qualitative research of the experiences of nurses in dementia care (shown in Table 2): “increased workload due to characteristics of dementia”, “increased mental stress”, “difficulty associated with playing a mediator role in addition to nursing duties”, and “lacking systematic support for dementia patient care”. Details of the synthesized themes are as follows.

### 3.1. Increased Workload Due to the Characteristics of Dementia

#### 3.1.1. The Unpredictable Nature of Dementia

Nurses in dementia care were under pressure from demanding workloads and time constraints [26,31,32]. As dementia care is time-consuming, nurses’ daily schedules were disrupted, and they experienced time shortages [32]. Addressing the pain of dementia patients was particularly time-consuming, and nurses were occasionally faced with difficulties as they could not obtain sufficient information from the patients [31]. BPSD was another factor that disrupted nurses’ occupational duties [29]. Nurses in dementia care experienced increased workload due to BPSD [24,25,29,33]. If patients displayed repetitive speech or behaviors, nurses began speaking or behaving repetitively as they did; as this led to increased workload, some nurses became angry [29].

#### 3.1.2. Ambiguous Communication with Dementia Patients

Nurses experienced difficulty with ambiguous communication from dementia patients [25,29,31,32,33]. Due to the difficulties in communication, nurses had trouble identifying dementia patients’ emotion or intent [29] and consequently found themselves in situations wherein they had no idea what they needed to do to satisfy their patients’ needs [29]. To communicate with dementia patients, nurses had to utilize various verbal and nonverbal communication strategies: speaking slowly, establishing eye contact, waiting, reading facial expressions, and using body language [25,31,32,33].

### 3.2. Increased Mental Stress

#### 3.2.1. Aggression in Dementia Patients

Nurses reported emotionally startling experiences resulting from their dementia patients’ symptoms [23,27]. They were confounded by the fact that they needed to understand their patients and establish emotional connections with them to provide appropriate care, despite having felt fear due to the aggression exhibited by those patients [23]. Others reported feeling skeptical toward the nursing profession when dementia patients constantly committed sexual harassment, which is another symptom of dementia [27]. Some nurses felt guilty when they had to take the second best option: for instance, lying to their patients to pacify dementia symptoms, forcibly suppressing their demands, unwillingly administering necessary medication, or defending themselves using barriers [26].

#### 3.2.2. Becoming More Apathetic

In addition, some nurses found themselves paying less attention to dementia patients when there were other patients who needed physical assistance [26]. Initially, nurses regretted such decisions; however, they eventually found themselves becoming habituated to such situations, observing that they were becoming emotionally numb [26]. Nurses reported that their occupational identity was at risk, as they were constantly exposed to events such as criticisms from guardians, delusion, aggression from elderly dementia patients, and death of their patients [26,27]. Through such experiences, nurses eventually treated elderly dementia patients solely from an occupational point of view—devoid of sympathy—and started losing respect for them as human beings [26]. As a result, nurses experienced stress [32].

### 3.3. Difficulty Associated with Playing a Mediator Role in Addition to Nursing Duties

#### 3.3.1. Mediator between Healthcare Professionals

As nurses know the most about their patients’ condition, it is crucial that they communicate information and cooperate with other healthcare professionals [25]. Nurses described their role as coordinators who were at the center of all work and, as professionals related to patients, knowing everything about the patients and capable of mediating on all matters [30]. In long-term care facilities without resident physicians, nurses found themselves under moral distress when they could not obtain the cooperation of their bi-monthly visiting doctor to examine a dementia patient whose health had deteriorated, as they were stuck in a situation without the authority to take a decision [26]. In cases where nurses had to play the role of doctors due to the latter’s unavailability, they were concerned about their ability to provide appropriate care for their patients [26]. Nurses were acting as the liaison between visiting doctors and elderly dementia patients and their guardians [26]. In this process, they suffered from emotional exhaustion, faced with the grievances expressed by all parties involved [26]. Further, when caregivers had difficulties with the elderly with dementia, nurses felt helpless that they are unable to speak for the rights of the caregivers [26].

#### 3.3.2. Mediator between Patient and Family Caregiver

Nurses also need to cooperate with the patients’ family members [25,32]. As it is the patients’ family members who provide information on the patient, nurses found it resourceful to cooperate with them to provide care for the patients [25,30,32]. However, there were cases wherein the family members could not comprehend the patients’ symptoms, becoming angry and emotional; in such situations, nurses had difficulty pacifying the family members, explaining that these were the symptoms of dementia [25].

### 3.4. Lacking Systematic Support for Dementia Patient Care

#### 3.4.1. Lack of Material Resources

Nurses were faced with the reality that it was difficult to provide sufficient care for dementia patients due to a lack of material resources [24,27]. Due to the unavailability of the necessary equipment for dementia patients’ care (e.g., professional rehabilitation therapy devices, appropriate palliative care devices), nurses felt as though they were pushing the limits of their patients’ safety [27]. Nurses were also angry from systematic factors that did not provide them with sufficient wages and necessary support [28].

#### 3.4.2. Lack of Human Resources

Nurses reported cases wherein there were no reinforcements in human resources, despite the increased demand for nursing hours due to the dementia patients’ symptoms [24]. Feeling the pressure from time constraints caused by a lack of manpower, nurses were frustrated by the reality where they had to prioritize “paperwork” over patient-centered care [28]. Moreover, the increased workload—due to the lack of manpower—made it difficult for nurses to focus on patient care; some reported experiencing guilt as they could not provide the best care for dementia patients [26]. Overall, nurses reported having felt extreme stress due to such factors [23,24,27].

#### 3.4.3. Lack of Educational Resources

Regarding the ceaseless wave of problems that appear in the process of nursing elderly dementia patients, who are quite unpredictable, nurses were learning to solve them without any guidance or resources [27]. Nurses reported the need for professional education, as professional knowledge was necessary to provide specialized care to dementia patients for different levels of symptom severity [24,27,28,30,32,33]. As the education that nurses had received in the past was at an elementary level, nurses in dementia care were seeking education for practical needs, such as dementia-related knowledge and methods of communication [28].

## 4. Discussion

The unpredictable nature of dementia patients, in combination with ambiguous communication, increased the workload of dementia care nurses. Due to the neurological symptoms of dementia that damage patients’ bodily and cognitive functions, they suffer from frequent falls [36], meaning that nurses need to pay continuous attention to them. As the unpredictable nature of dementia inevitably leads to increased workload and frustration, nurses feel pressured from overburden and time constraints [9]. Therefore, there is a need for a computational method to calculate the changes in nurses’ workload according to the severity of the patients’ dementia symptoms, so that nurses can provide sufficient care to their patients without feeling pressured from their duties. Nurses also had difficulty communicating with dementia patients showing symptoms of aphasia. When communication between nurses and their patients decreases, the BPSD of dementia patients worsens, as nurses have no means of identifying their patients’ emotions or needs [37]. Thus, nurses felt more pressure from providing dementia care [37]. Therefore, there is a need to develop educational programs that teach nurses dementia-specific communication strategies to communicate effectively with their patients. If nurses were to become capable of communicating effectively with their dementia patients through such programs, it would alleviate the problems of heavy workload and increased stress.

Nurses also suffered from increased mental stress when they were providing care for dementia patients. Nurses who have suffered injuries from aggressive dementia patients faced emotional challenges [38] and also experienced high levels of distress [39]. Nurses felt fear and frustration when they were verbally abused, sexually harassed, or physically attacked by dementia patients, and were uncomfortable as they did not feel respected as nurses [40]. After suffering from such incidents, nurses faced an existential crisis toward their occupation, while also experiencing high levels of stress [40]. Nurses also started treating elderly dementia patients in an apathetic and strictly professional manner, having difficulty treating and respecting them as human beings. Nurses would communicate very briefly with dementia patients when they needed to fulfill their patients’ daily physical needs (e.g., distributing meals, checking medical charts, organizing); sometimes, they would not communicate at all with their patients [41]. Therefore, to protect nurses who have been physically or psychologically abused by their dementia patients with BPSD, there is a need to establish some systematic means of providing safety measures in such cases. Specific types of devices would be required, such as a safety alarm device [10]. In addition, nurses need support to reduce the stress they receive from their dementia patients’ symptoms, such as counseling sessions or mindfulness programs. If nurses’ physical and psychological suffering could be reduced through such measures, they will be able to provide high-quality care to their dementia patients.

Nurses in dementia care felt that their role as mediators was crucial for their work, but they also felt the difficulty of playing that role. Nurses in long-term care facilities are in charge of managing and overseeing numerous healthcare staff (e.g., nurse assistants) in addition to their clinical duties [42]. In such environments, sharing knowledge with doctors, occupational therapists, and physical therapists is a necessary condition for providing high-quality care. The results of the current study indicate that nurses feel anxious and frustrated when doctors’ capabilities and knowledge are insufficient. Nurses need to play a mediator role not only between medical professionals, but also between dementia patients and their family members. Occasionally, nurses experience difficulty handling family members and guardians, and they feel pressured in communicating with them due to workloads and time constraints [43]. Creating a good relationship with dementia patients’ family members is a prerequisite of high-quality care [44], and nurses need to enable the family members’ involvement in their patients’ lives, as patients have a long-lasting relationship with their family. As dementia patients have limited communication skills due to their symptoms, creating a good relationship with family members is also important to obtain information that would otherwise be unavailable [45]. The mediator role played by nurses has not been included in estimating their workloads. As nurses have difficulty playing these mediator roles, a measure for quantifying and accommodating such commitments, as part of their workload, is necessary.

Importantly, nurses are not receiving the appropriate systematic support they need to provide high-quality care for their dementia patients. Lack of resources is another factor that hinders nurses from providing appropriate care [44]. The hospital environment also affected nurses’ work: when the physical environment of a hospital was narrow and confined, dementia patients became more hypersensitive and irritable [46]. On the contrary, nurses working in hospitals with comfortable physical environments reported high work efficiency and job satisfaction, as communication was made easier for them and their dementia patients [46]. Organizations that pushed for cost-effectiveness made nurses feel pressure when providing the necessary medical measures to their patients [47]. In a study of 687 nursing home nurses, 48% of the nurses were dissatisfied with their wage levels [48]. It is crucial to have sufficient nursing personnel in order to have meaningful communication with dementia patients and be able to provide them appropriate end-of-life care [44]. As dementia patients behave slowly, nurses require sufficient time to be able to provide adequate care [46]. Research has found that increasing a nurse’s hours per resident day (HPRD) spent on their dementia patients by a mere two minutes led to an 8% increase in the quality of care outcomes [49], which implies the need for increasing the number of nursing staff. As the personnel working in dementia care experience poor working conditions, they have insufficient education and training, and do not have the opportunity to advance their careers [50]. As for hospital nurses, only 40% of them had received education and training for dementia [51]. Even in professional care facilities specializing in dementia, most of the nurses lacked expertise in dementia care, which led to even larger burdens on workloads [44]. Therefore, to help provide the best care for their dementia patients, systematic regulations should be established to provide nurses with physical resources, manpower, and educational support at an institutional level.

This study has some limitations. First, the findings in this study are based on studies conducted in five countries (United Kingdom, Ireland, South Korea, Taiwan, and Sweden), therefore our results may not be possible to generalize in worldwide nurses. Second, we evaluated selected articles using the CASP checklist, we found that some articles may have an unclear risk of bias. Therefore, taking into methodological limitation, our findings should be interpreted carefully. Finally, we only included articles written in English and Korean. Therefore, we may have missed other articles related to this study. Despite these limitations, the significance of the current study lies in its comprehensive analysis of dementia care nurses’ workloads and mental stress that identified foundational data that could be used to improve nurses’ work environments and care quality. As nurses’ workloads and mental stress in dementia care should be considered and managed efficiently, future thorough investigations, where the concrete measurements of nursing workloads in dementia care are invented, are required. Particularly, this study would contribute to disparities in issues of dementia care, a typical area of vulnerable populations, by exploring and synthesizing the nursing work-related issues from nurses’ experiences caring with dementia patients.

## 5. Conclusions

Although the mediator role played by nurses is of critical importance, this study highlights that nurses have difficulty playing that role. This study has verified that nurses do not have enough support to provide sufficient care for their patients. There is a need to develop a method for estimating nurses’ workloads, considering the varying characteristics of dementia patients. Nurses also need support to heal from the emotional burden they experience from their dementia patients, such as counseling sessions or mindfulness programs. To help nurses perform better in their role as a mediator, they need both individual-level support (e.g., providing education on communication strategies) and institutional-level support (e.g., resolving systematic issues). Finally, the working conditions of nurses in dementia care should be improved, along with providing additional resources. In conclusion, healthcare disparity in providing dementia care compared with the other populations’ care was identified throughout this meta-synthesis. Exploring nurses’ workload-related issues in dementia care and vulnerable population care is meaningful and necessary in providing appropriate care to older adults with dementia, and will contribute to resolve disparities of caring individuals with dementia.

## Figures and Tables

**Table 1:** Summary of the included articles.

Reference Number	Participants (Country)	Data Collection Method	Analysis Method	Theme Reported	CASP Score
[23]	8 nurses (United Kingdom)	Semi-structured interviews	Interpretative Phenomenological Analysis (IPA)	Effort to sense make; Pressures of the organization; Balancing personal and professional selves: The underlying emotional connection; Looking back on it.	9/10
[24]	9 nurses (Ireland)	Semi-structured interviews	Elo and Kyngas framework	Recognizing and understanding responsive behavior; Resources and interventions to support people with dementia and responsive behavior; The impact of education on nursing practice; The care environment.	9/10
[25]	13 nurses (South Korea)	In-depth interviews	Grounded theory	Caring feelings from the heart; Maturity as a nurse; Understanding care of older adults; Unconditional caring; Familial support; Confidence in nursing; Building trust with clients; Implementing emotional care; Caring for dementia symptoms; Collaborating with human resources; Gratification in nursing practice; Appreciation toward life.	8/10
[26]	12 nurses (South Korea)	Semi-structured interviews	Colaizzi’s phenomenological approach	Worrying about sincere care; A sense of guilt about task-oriented care; A feeling of helplessness as they cannot advocate care.	8/10
[27]	7 nurses (South Korea)	In depth interview	Colaizzi’s phenomenological approach	Accepting dementia as a part of life; Crumbling sense of identity due to the provocation of elders with dementia and their families; Pursuing caring beyond dementia caring.	8/10
[28]	11 nurses (United Kingdom)	Focus group interviews	Thematic analysis	Responsibilities and frustrations; It’s Not Like the NHS (National Health Service); Barriers to Learning; Future Training.	8/10
[29]	15 nurses (Taiwan)	In-depth interviews	Conventional content analysis	Different language; Blocked messages.	8/10
[30]	10 nurses (South Korea)	In-depth interviews	Colaizzi phenomenological method	New encounter; Understanding of intention and satisfying; Dilemma of caring; Contriving of comfort; Being present.	8/10
[31]	24 registered nurses (Sweden)	Self-reported questionnaire form	Graneheim and Lundman’s qualitative content analysis approach	Non-verbal communication; The pain assessment; Advice and suggestions for improvement.	6/10
[32]	21 nurses (Sweden)	Focus group interviews	Qualitative content analysis	Communication; Visual assessment of pain; Practical issues.	6/10
[33]	51 nurses (Sweden)	Self-administered questionnaire (23 items)	Qualitative content analysis	Visual assessment of pain; Communication.	5/10

**Table 2:** Original study themes which contributed to the final meta-synthesis themes.

Themes	[23]	[24]	[25]	[26]	[27]	[28]	[29]	[30]	[31]	[32]	[33]
1. Increased workload due to the characteristics of dementia -The unpredictable nature of dementia		V	V	V			V		V	V	V
-Ambiguous communication with dementia patients			V				V		V	V	V
2. Increased mental stress -Aggression in dementia patients	V			V	V						
-Becoming more apathetic				V	V				V		
3. Difficulty associated with playing a mediator role in addition to nursing duties -Mediator between healthcare professionals			V	V				V			
-Mediator between patient and family caregiver			V					V	V		
4. Lacking systematic support for dementia patient care -Lack of material resources		V			V	V					
-Lack of human resources	V	V		V	V	V		V	V		V
-Lack of educational resources		V			V	V		V		V	V

## Data Availability

All the included studies are in Table 1.
